# Contralateral lymph node metastasis in recurrent ipsilateral breast cancer with Lynch syndrome: a locoregional event

**DOI:** 10.1186/s12957-023-02918-w

**Published:** 2023-02-09

**Authors:** Tibor A. Zwimpfer, Fabienne D. Schwab, Daniel Steffens, Felix Kaul, Noemi Schmidt, James Geiger, Franziska Geissler, Viola Heinzelmann-Schwarz, Walter P. Weber, Christian Kurzeder

**Affiliations:** 1 Peter MacCallum Cancer Centre, Melbourne, Australia; 2 Department of Obstetrics and Gynaecology, University Hospital of Basel, 4056 Basel, Switzerland; 3 Breast Centre, University Hospital Basel, Basel, Switzerland; 4 Department of Radiology and Nuclear Medicine, University Hospital Basel, Basel, Switzerland

**Keywords:** Breast neoplasms, Recurrence, Lynch syndrome, Lymphoscintigraphy, Sentinel lymph node biopsy

## Abstract

**Introduction:**

Contralateral axillary lymph node metastasis (CALNM) in breast cancer (BC) is considered a distant metastasis, marking stage 4cancer. Therefore, it is generally treated as an incurable disease. However, in clinical practice, staging and treatment remain controversial due to a paucity of data, and the St. Gallen 2021 consensus panel recommended a curative approach in patients with oligometastatic disease. Aberrant lymph node (LN) drainage following previous surgery or radiotherapy is common. Therefore, CALNM may be considered a regional event rather than systemic disease, and a re-sentinel procedure aided by lymphoscintigraphy permits adequate regional staging.

**Case report:**

Here, we report a 37-year-old patient with Lynch syndrome who presented with CALNM in an ipsilateral relapse of a moderately differentiated invasive ductal BC (*ER* 90%, *PR* 30%, HER2 negative, Ki-67 25%, microsatellite stable), 3 years after the initial diagnosis. Lymphoscintigraphy detected a positive sentinel LN in the contralateral axilla despite no sign of LN involvement or distant metastases on FDG PET/CT or MRI. The patient underwent bilateral mastectomy with sentinel node dissection, surgical reconstruction with histological confirmation of the CALNM, left axillary dissection, adjuvant chemotherapy, and anti-hormone therapy. In addition to her regular BC follow-up visits, the patient will undergo annual colonoscopy, gastroscopy, abdominal, and vaginal ultrasound screening. In January 2023, the patient was free of progression for 23 months after initiation of treatment for recurrent BC and CALNM.

**Conclusion:**

This case highlights the value of delayed lymphoscintigraphy and the contribution of sentinel procedure for local control in the setting of recurrent BC. Aberrant lymph node drainage following previous surgery may be the underlying cause of CALNM. We propose that CALNM without evidence of systemic metastasis should be considered a regional event in recurrent BC, and thus, a curative approach can be pursued. The next AJCC BC staging should clarify the role of CALNM in recurrent BC to allow for the development of specific treatment guidelines.

## Introduction

Contralateral axillary lymph node metastasis (CALNM) in breast cancer (BC) is a condition with a rare incidence of 1.9–6% [1–5]. CALNM can occur as a metastasis of the primary BC or a different extramammary primary cancer or with an occult ipsilateral BC as an origin [6–8]. Metastasis of the primary BC is the most common cause. It can be detected upon the first diagnosis of the primary BC or at relapse of the BC. Regardless of the origin of CALNM, any metastases of BC to the contralateral axillary lymph nodes are considered to be a distant metastasis, marking stage 4 of cancer, according to the American Joint Committee on Cancer (AJCC) staging system [9]. Considering CALNM as a metastatic disease has profound implications on locoregional management. In clinical practice, staging and treatment of CALNM remain controversial due to lack of evidence especially in the recurrent setting after surgery.

Aberrant lymph node drainage following previous surgery or radiotherapy is commonly described with 18–70% in the literature [10–12]. Therefore, CALNM may be considered a regional event rather than systemic disease, and a re-sentinel procedure aided by lymphoscintigraphy permits adequate regional staging [5, 10, 13–16]. Here, we present a premenopausal Lynch syndrome patient with ipsilateral relapse of invasive ductal BC and CALNM detected by a re-sentinel procedure and subsequent treatment with curative intent.

## Results

### Patient presentation

In April 2017, a 34-year-old premenopausal primipara woman underwent oncoplastic resection with sentinel biopsy on the right side with diagnosis of a poorly differentiated invasive ductal BC. The lesion was staged as pT1c (15 mm), pN1mi (1/2) (sentinel node (sn)), Bloom–Richardson–Elston (BRE) grade 3, R0, estrogen receptor (ER) 90%, progesterone receptor (PR) 60%, human epidermal growth receptor 2 (HER2) negative, and Ki-67 15%. The initiated oncotype DX showed a score of 18. The patient received adjuvant radiotherapy to the right breast and axilla (50.4 Gray (Gy)), paraclavicular (45 Gy), and a local boost dose of (6 × 2 Gy). Concomitantly, antihormonal therapy with tamoxifen was initiated; however, due to adverse effects (menopause-like symptoms, weight gain, and exacerbation of her depression), treatment was discontinued by the patient after 2 months without further antihormonal treatments.

Genetic counseling and testing showed a familial predisposition towards endometrial, liver, kidney, and lung cancer. A verified Lynch syndrome mutation was identified in the patient’s mother in 2015 and was subsequently confirmed in the patient with diagnosis of the poorly differentiated invasive ductal BC; it is a heterozygous mutation of the mismatch repair gene MSH6: c.2690dupA/p.Asn897LysfsTer3. As a result, the patient opted for a preventive hysterectomy and salpingectomy after completing family planning.

The patient’s medical history included three deep vein thromboses secondary to a hereditary antithrombin deficiency type 1. Her medical history also included a vaginal birth in 2005, newly diagnosed depression and hyperthyroidism in 2020, and 20-pack-year smoking history. Her current medications included Eliquis (5 mg PO, twice a day), escitalopram (20 mg PO once a day), and Tirosint (25 μg PO once a day).

**Fig. 1:**
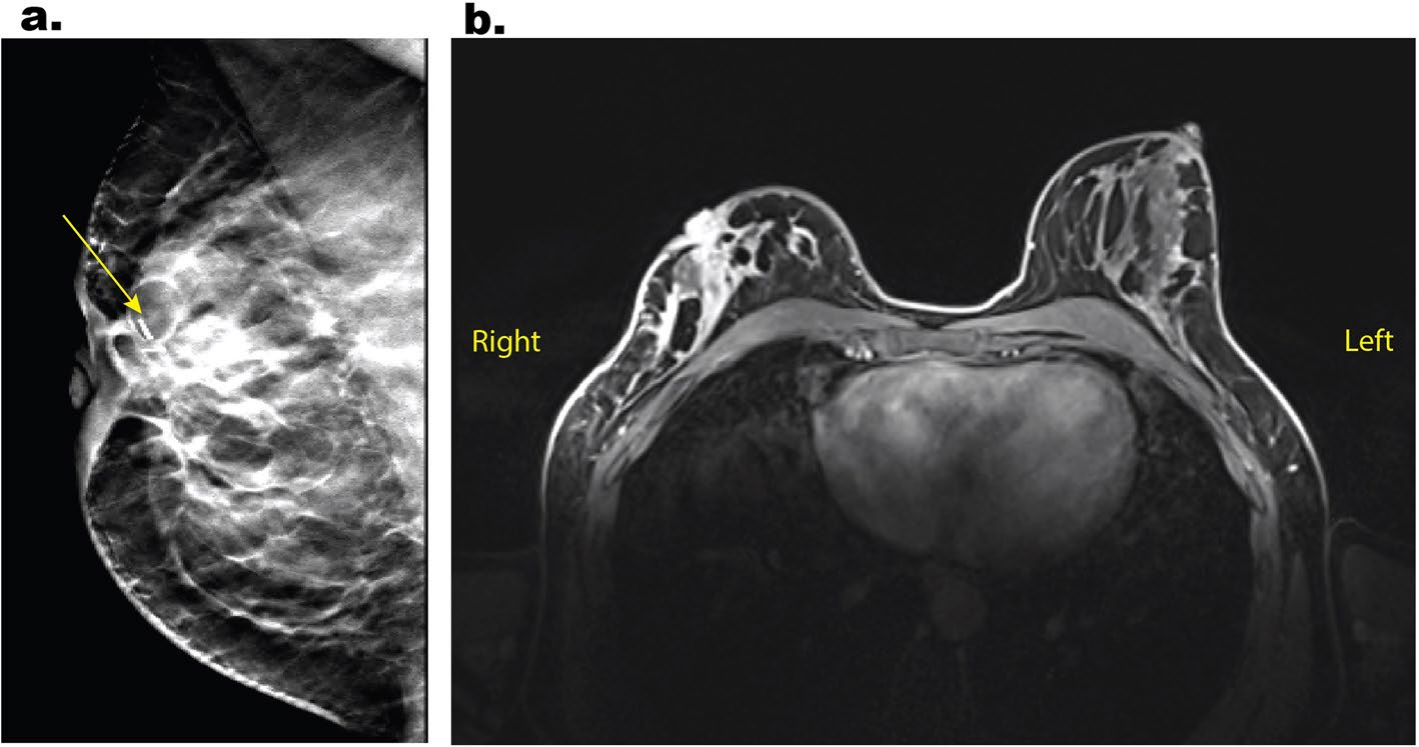
Mammogram and magnetic resonance imaging (MRI) showing ipsilateral breast cancer relapse before neoadjuvant antihormone therapy. **a** Mammogram of the right breast in a mediolateral oblique view with suspicion of a relapse BIRADS 4, ACRb. It shows increased parenchymal density of the upper right quadrant compared to the previous follow-ups, progressive microcalcifications medial to the clips (arrow), and progressive mamilla retraction. **b** T1-weighted transverse axial dynamic contrast-enhanced MRI with fat suppression showing asymmetric accumulation of contrast and restricted diffusion of the whole right breast including the mammilla. There is subcutaneous edema of the right breast, but no sign of lymph node metastases or distant metastases

The follow-up mammogram in December 2020, 3 years after the initial diagnosis, revealed increased parenchymal density and progressive micro-calcifications in the right breast compared to the regular previous imaging. These findings were suspicious for an ipsilateral relapse of invasive ductal BC (Fig. 1).

The biopsy confirmed a relapse with infiltrates of a moderately differentiated invasive ductal BC (*ER* 90%, *PR* 30%, HER2 negative, Ki-67 25%, microsatellite stable). The patient was started preoperatively on an endocrine therapy with the nonsteroidal aromatase inhibitor (AI) letrozole (2.5 mg PO QD) along with the gonadotropin-releasing hormone agonist goserelin (3.6 mg SQ QM) for ovarian function suppression (OFS).

**Fig. 2:**
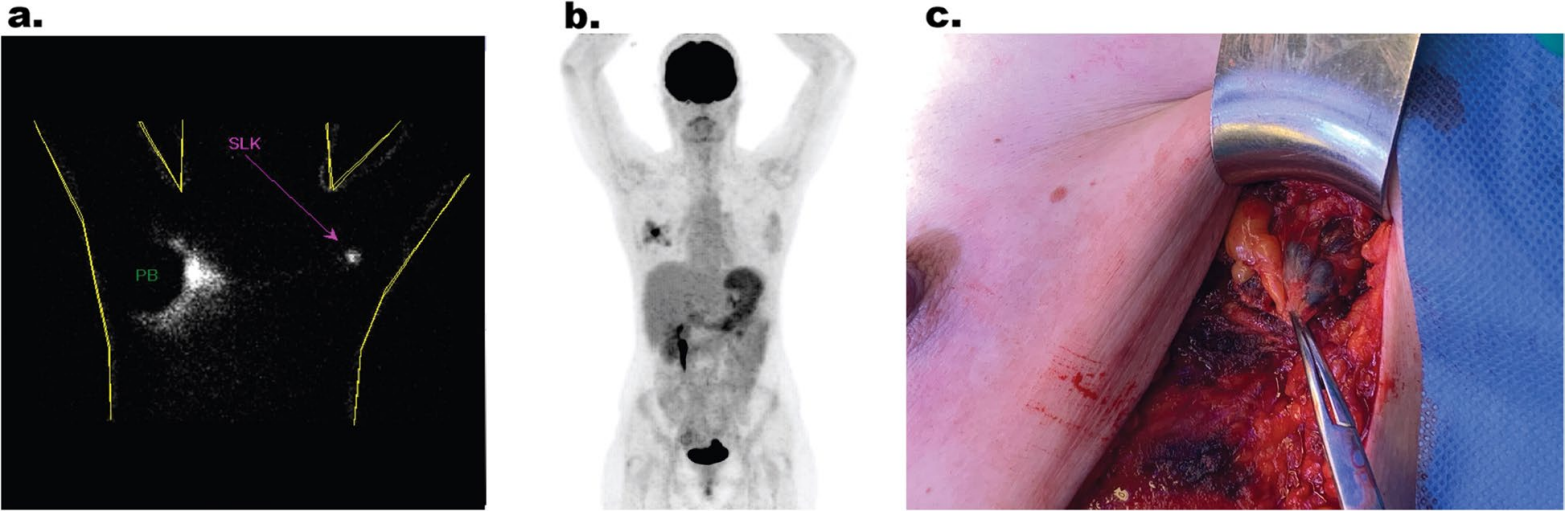
Contralateral lymph node metastasis in recurrent ipsilateral breast cancer. **a** Planar sentinel node scintigraphy 15 min after periareolar injection of 70 MBq ^99m^Technetium-labelled nanocolloid (NanoHSA-ROTOP®), with detection of the sentinel node (pink arrow) in the left axilla (PB = injection site covered with lead). **b** Maximum intensity projection of the FDG PET/CT scan, demonstrating diffuse multifocal hypermetabolism of the recurrent mamma carcinoma on the right, without lymph node metastases or distant metastases. **c** Intraoperative left axilla with detection of two patent blue V and technetium-positive lymph nodes

Preoperative evaluation with fluorodeoxyglucose positron emission tomography with computed tomography (FDG PET/CT) and magnetic resonance imaging (MRI) confirmed relapse of the right breast, with no sign of lymph node involvement or distant metastases. However, delayed lymphoscintigraphy (2 h after injection) detected a sentinel lymph node in the contralateral axilla (Fig. 2).

### Surgical and medical management

The management options were discussed at the interdisciplinary tumor board and with the patient, and it was decided to pursue a curative approach with re-sentinel procedure.

The patient underwent bilateral mastectomy with sentinel lymph node dissection and surgical reconstruction. In detail, the procedure included the following: (a) skin-sparing mastectomy with thoracodorsal artery perforator flap and implant reconstruction on the right, (b) nipple-sparing mastectomy with implant insertion on the left, and (c) sentinel lymph node dissection bilaterally. During the sentinel node dissection, three technetium and patent blue lymph nodes were identified in the contralateral axilla and removed (Fig. 2).

Histopathological analysis confirmed the presence of an ipsilateral invasive ductal tumor and contralateral lymph node metastases. The patient was staged as rpT3 (96mm), rpN1a (2/3, contralateral) (0/4, ipsilateral), G2, V0, L1, Pn1, R0 (local), *ER* 90%, *PR* 30%, HER2 negative, and Ki-67 5%. The following axillary dissection on the left after 4 weeks resulted in no additional positive lymph nodes rpN1a (2/18).

Four days later, the patient returned to the hospital with a ruptured ovarian cyst and ovarian torsion on the right. She underwent right adnexectomy and was discharged on postoperative day 2.

The OFS with goserelin was continued, and adjuvant chemotherapy with 6 cycles of Taxotere and cyclophosphamide (TC) was initiated. After three cycles of TC, she developed methicillin-resistant *Staphylococcus aureus* cellulitis of the right arm secondary to chemotherapy-induced neutropenia. The cellulitis was treated with incision and drainage, followed by 14 days of IV daptomycin and piperacillin/tazobactam. Thereafter, the remaining 3 cycles of TC were well tolerated without adverse effects.

After completing chemotherapy, endocrine therapy with AI was resumed. Additionally, the patient received the bisphosphonate zoledronic acid (4 mg IV q6month) and underwent regular bone density measurements. In addition to her regular BC follow-up visits, the patient will undergo annual colonoscopy, gastroscopy, abdominal ultrasound screening, and vaginal ultrasound screening. As of August 2022, the patient has been free of progression for 23 months after initiation of treatment for recurrent BC with CALNM until now.

### Literature review

In the literature, 49 histologically confirmed CALNM with ipsilateral BC recurrence have been described (Table 1). The literature consists of 7 case reports, 4 small retrospective series, and 5 prospective repeat sentinel biopsy (SNB) studies. Thirty-five of 49 patients were treated with a curative approach, and the therapeutic approach of the remaining 14 patients was not reported. Five of these patients had micrometastases (< 2 mm). SNB was applied in 36.7% (18/49) of the patients and preoperative lymphoscintigraphy in 40.8% (20/49), respectively, with 14 not reported for both methods.

**Table 1:** Review of the literature related to ipsilateral breast cancer recurrence with histologically confirmed contralateral lymph node metastasis

Author	Year	Article type	Number	LSG	SNB	Treatment
**Lim I. et al.** [13]	2004	Case report	1	Yes	Yes	Curative
**Agarwal A. et al.** [14]	2005	Prospective repeat SNB study	1	Yes	Yes	Curative
**Roumen R. et al.** [11]	2006	Prospective repeat SNB study	2	Yes	Yes	Curative
**Taback B. et al.** [15]	2006	Prospective repeat SNB study	2	Yes	Yes	Curative
**Tasevski R. et al.** [16]	2009	Retrospective case series	1	Yes	Yes	Curative
**Maaskant-Braat A. et al.** [5]	2013	Prospective repeat SNB study	5	Yes	Yes	Curative
**Nishimura S. et al.** [17]	2014	Case report	1	Yes	Yes	Curative
**Tokmak H. et al.** [18]	2014	Prospective repeat SNB study	1	Yes	Yes	Curative
**Wang W. et al.** [19]	2014	Retrospective case series	14	NA	No	NA
**Chkheidze R. et al.** [20]	2017	Retrospective case series	2	Yes	Yes	Curative
**Strazzanti A. et al.** [4]	2018	Case report	1	Yes	No	Curative
**Magnoni F. et al.** [21]	2020	Retrospective case series	14	No	NA	Curative
**Herrera-Martinez Y. et al.** [22]	2021	Case report	1	Yes	Yes	Curative
**Maseki H. et al.** [23]	2021	Case report	1	Yes	No	Curative
**Salih A. et al.** [24]	2021	Case report	1	No	No	Curative
**Goh IY. et al.** [25]	2022	Case report	1	Yes	Yes	Curative
**Current study**		Case report	1	Yes	Yes	Curative

*LSG*, lymphoscintigraphy; *SNB*, sentinel lymph node biopsy; *NA*, not applicable

## Discussion

Typically, in BC management, a positive contralateral lymph node would be classified as distant site metastasis [9]. However, aberrant lymph node drainage following previous surgery or radiotherapy is common. Alternative routes of lymph node drainage develop because of damage to the usual lymphatic system [10–12, 15, 26–30]. This can be caused by previous breast and axillary surgery or irradiation for primary BC [13, 30–32].

Therefore, if an ipsilateral BC recurrence is diagnosed, the physical examination and imaging with ultrasound, MRI, and FDG PET/CT can be helpful in detecting a CALNM [33]. Furthermore, delayed lymphoscintigraphy should be considered and can be crucial in the setting of tumor recurrence and expected aberrant lymphatic drainage. As in our case, the FDG PET/CT and MRI showed no signs of lymph node metastasis, whereas the lymphoscintigraphy showed a Tc99m signal in a contralateral lymph node (Fig. 2). Subsequent surgical staging by sentinel procedure was performed. In contrast to FDG PET/CT, the sentinel procedure can detect small volume metastatic disease and may therefore add valuable information for adjuvant treatment decisions and improve regional control [34].

Although there is still a lack of guidelines and consensus on CALNM treatment in the recurrent setting, most patients with ipsilateral breast cancer recurrence and CALNM were treated with a curative approach (Table 1). The curative approach is supported by Moossdorff et al. [26] demonstrating that the prognosis of CALNM (82.6% overall survival after mean 50.3 months) receiving locoregional and systematic treatment in the majority of cases is significantly better than the prognosis of patients with metastatic BC and is in line with the prognosis of patients with a regional recurrence. Therefore, a curative treatment approach for patients with CALNM in recurrent BC may have similar outcomes to those with locally advanced stage 3B BC and could be reclassified as ipsilateral supraclavicular disease from M1/stage 4 to N3c/stage 3C in the 6th edition of the *AJCC Cancer Staging Manual* [35]. However, prospective trials are needed to validate the results of these case reports and retrospective case series.

Taken together, in the recurrent setting, we consider contralateral lymph node metastasis to be a regional event, and therefore, efforts for early detection should be included in the diagnostic workup to allow for treatment with curative intent. In our case, the patient underwent left axillary dissection, adjuvant chemotherapy, and anti-hormone therapy. A non-anthracycline regimen with 6 cycles of TC was used since there was an increased risk of cardiac complications because of the patient’s history of deep vein thromboses and hereditary antithrombin deficiency.

Based on the literature review provided, the incidence of CALNM in recurrent breast cancer cannot be estimated. Patients presenting with CALNM are usually young, with aggressive histopathological features, and an altered lymphatic spread supports the development of CALNM [21, 36, 37]. Overall, BC in young women is characterized by a higher proportion of basal-like, triple-negative, and HER2-enriched tumors, which are often poorly differentiated with lymphovascular invasion [38]. Additionally, young age is an independent risk factor for increased local recurrence [39, 40].

This patient was 37 years old at relapse with luminal B-like features (*ER* 90%, *PR* 30%, and Ki-67 25%) and no HER2 overexpression. Additionally, a pathologic mutation of the mismatch repair gene *MSH6* is known, which is associated with microsatellite instability. Lynch syndrome, also called hereditary nonpolyposis colorectal cancer, is an autosomal dominant inherited disorder with changes in the *MLH1*, *MSH2*, *MSH6*, *PMS2*, or *EPCAM* gene that increases the risk of various cancer types [41].

For women, there is an increased lifetime risk for colorectal carcinoma (30–45%), endometrial cancer (25–50%), and ovarian cancer (6–14%) with Lynch syndrome [42]. The current data for the risk of BC with Lynch syndrome are inconclusive [42–44]. But it has been shown that *MSH6* is associated with a 31% [45] higher risk and odds ratio of 1.65 (95% *CI*, 1.06 to 2.52) [46] for BC. In contrast, the BC relapse in our patient showed no microsatellite instability. Because of the high-risk profile of Lynch syndrome, our patient is scheduled for annual cancer screening, and the removal of the remaining ovary may be an option and would allow to continue the AI without OFS.

## Conclusion

The rarity of CALNM in BC relapse, along with the controversial staging, complicates the therapeutic decision-making process. This case highlights the value of delayed lymphoscintigraphy and the contribution of sentinel procedure for local control in the setting of recurrent BC. We propose that CALNM without evidence of systemic metastasis should be considered a regional event in recurrent BC, and thus, a curative approach can be pursued. The next AJCC BC staging should clarify the role of CALNM in recurrent BC and adjust the treatment guidelines accordingly.

## Data Availability

The data and materials analyzed for the current case report are presented within the manuscript and are also available from the corresponding author. Clinicopathological patient data used during this study are stored in a privacy-compliant locked database and cannot be made available to protect patient privacy.

## Abbreviations

CALNM: Contralateral axillary lymph node metastasis
BC: Breast cancer
LN: Lymph node
FDG PET/CT: Fluorodeoxyglucose positron emission tomography with computed tomography
MRI: Magnetic resonance imaging
SNB: Sentinel lymph node biopsy
L: Lymphoscintigraphy
